# Immediate management of a cirrhosis-induced severe pericardial effusion: a case report and review of the literature

**DOI:** 10.1186/s13256-024-05016-x

**Published:** 2025-01-08

**Authors:** Maryam Taheri, Arash Hassanpour Dargah, Pedram Ramezani, Mohsen Anafje, Amir Nasrollahizadeh, Pouya Ebrahimi, Mohammad Hossein Mandegar

**Affiliations:** 1 Tehran Heart Center, Cardiovascular Disease Research Institute, Tehran University of Medical Sciences, Tehran, Iran; 2 School of Medicine, Alborz University of Medical Sciences, Karaj, Iran; 3 Endocrinology and Metabolism Research Center, Tehran University of Medical Sciences, Tehran, Iran; 4 Rajaei Cardiovascular Medical and Research Rajaie Cardiovascular Medical and Research institute, School of Medicine, Iran University of Medical Sciences, Tehran, Iran; 5 Cardiac Surgery Department, Imam Khomeini Hospital, Tehran University of Medical Sciences, Tehran, Iran

**Keywords:** Tamponade, Liver cirrhosis, Autoimmune hepatitis, Pericardial effusion, Systemic inflammation

## Abstract

**Introduction:**

Cardiac tamponade is a life-threatening condition resulting from fluid accumulation in the pericardial sac, leading to decreased cardiac output and shock. Various etiologies can cause cardiac tamponade, including liver cirrhosis, which may be induced by autoimmune hepatitis. Autoimmune hepatitis is a chronic inflammatory liver disease characterized by interface hepatitis, elevated transaminase levels, autoantibodies, and increased immunoglobulin G levels. This case report details a 60-year-old male with autoimmune hepatitis-induced cirrhosis presenting with severe pericardial effusion and cardiac tamponade, emphasizing the interplay between liver and cardiac pathologies.

**Methods:**

A 60-year-old Persian man presented with progressive dyspnea, chest pain, and significant weight gain due to fluid retention. Physical examination revealed pallor, jaundice, elevated jugular venous pressure, muffled heart sounds, and tachycardia. Laboratory tests indicated severe hepatic and renal dysfunction, with elevated liver enzymes, bilirubin, and blood urea nitrogen. Imaging studies, including electrocardiogram, computed tomography angiography, and transthoracic echocardiogram, confirmed large pericardial effusion with signs of cardiac tamponade. Emergency pericardiocentesis was performed, aspirating 500 mL of serosanguinous fluid. Post-procedural management included continuous monitoring, repeat echocardiography, and a comprehensive pharmacological regimen addressing fluid overload, autoimmune hepatitis, and cardiac function.

**Conclusion:**

This case underscores the importance of timely diagnosis and management of cardiac tamponade, particularly in patients with concomitant conditions like autoimmune hepatitis and cirrhosis. Multidisciplinary management involving hepatologists, cardiologists, and critical care specialists is crucial for improving patient outcomes. Early recognition and treatment contribute substantially to the prevention of recurrence and better long-term management of underlying conditions.

## Clinical key points

Prompt recognition and intervention are critical for life-threatening cardiac tamponade. Autoimmune hepatitis can lead to severe complications, including cirrhosis and pericardial effusion. A multidisciplinary approach involving collaboration among cardiothoracic surgeons, interventional cardiologists, and other specialists is essential for managing complex cases involving multiple organ systems. Continuous monitoring and long-term management of autoimmune hepatitis and cirrhosis are crucial to prevent recurrence and improve patient outcomes.

## Introduction

Cardiac tamponade is a critical condition where fluid buildup in the pericardial sac exerts pressure on the heart, leading to reduced cardiac output and potentially causing shock [1]. Cardiac tamponade can arise from numerous causes, which are generally categorized as common and uncommon [2]. Liver cirrhosis is one of the etiologies that can lead to pericardial effusion [3], and one of the causes of liver cirrhosis is autoimmune hepatitis [4]. Also, it can cause cardiac tamponade through the development of hepatic hydrothorax and pericardial effusion due to severe portal hypertension and fluid imbalance. These effusions can lead to the compression of the heart, resulting in tamponade [5]. Epidemiologically, pericardial effusion occurs in approximately 63% of patients with ascites secondary to hepatic cirrhosis, compared with 11% in control subjects [6]. Given the pathophysiological mechanisms linking liver disease and cardiac complications, such as increased portal pressure and systemic inflammation, it is plausible that autoimmune hepatitis, through its progression to liver cirrhosis, can precipitate significant pericardial effusion leading to cardiac tamponade [7, 8].

Autoimmune hepatitis (AIH) is an inflammatory liver disease identified by histological interface hepatitis, elevated transaminase levels, and the presence of autoantibodies with increased immunoglobulin G (IgG) levels [9]. The global incidence and prevalence of AIH vary significantly, with pooled estimates showing an incidence of 1.28 cases, and a prevalence of 15.65 cases, per 100,000 people. These rates are higher in countries with a high Human Development Index, especially in North America and Oceania, and are more common among females and older adults [10]. About 30–50% of patients with autoimmune hepatitis develop liver cirrhosis, either at the time of diagnosis or during the disease course [11]. Clinical manifestations vary, and diagnosis is based on excluding other liver disorders and recognizing a suggestive clinical pattern [12].

This case report describes a 60-year-old male with a history of AIH and resultant severe cirrhosis and tamponade. Reporting this rare coincidence highlights the need for a holistic approach to the diagnosis and management of patients that present with secondary tamponade.

## Case presentation

A 60-year-old Persian male presented to the emergency room (ER) with worsening dyspnea, chest pain, and restlessness, which had started about 30 hours ago and had progressively worsened. He also reported swelling in his lower extremities but had no history of fever, cough, respiratory diseases, or heart problems. He had a past diagnosis of autoimmune hepatitis (AIH) leading to severe cirrhosis. The patient denied any recent travel, sick contacts, or changes in medication. He reported a significant weight gain (4 kg) over the past few weeks, mainly due to fluid retention. His medical history was notable for AIH, diagnosed 5 years ago, for which he had been on immunosuppressive therapy. He had no known allergies and had a family history of liver disease. Socially, he had a history of moderate alcohol consumption but quit drinking 5 years ago upon diagnosis. He had also smoked a pack of cigarettes a day for 20 years, but had stopped 10 years ago. He denied any illicit drug use.

Upon arrival, during the general examination, the patient appeared anxious and uncomfortable, with skin signs of chronic liver disease, including spider angiomas and palmar erythema. The patient was conscious and responsive, with the following vital signs: blood pressure (BP) 110/70 mmHg, heart rate (HR) 110 bpm, respiratory rate (RR) 22 breaths per minute, temperature (T) 37 °C, and oxygen saturation 95% on ambient air. Physical examination revealed pallor and jaundice. Cardiovascular examination showed elevated jugular venous pressure (JVP) of 15 cm H_2_O, muffled heart sounds (positive Beck’s triad), and tachycardia. The respiratory examination noted bilateral tachypnea without wheezing or crackling, and clear breath sounds. The abdominal examination indicated no ascites, but the liver span was palpable below the costal margin, with no splenomegaly. The extremities showed a 3+ pitting edema in the lower extremities. Neurological examinations revealed no significant abnormalities, with intact cranial nerves II-XII, no focal deficits, and normal reflexes and motor strength.

## Methods

Laboratory results revealed severe renal and hepatic dysfunction. The patient’s results showed severe hyperglycemia and renal impairment, as indicated by elevated blood urea nitrogen (BUN) levels. Liver function tests (LFT) revealed marked hepatic dysfunction with elevated liver enzymes and total bilirubin levels. Hematological findings highlighted leukocytosis with predominant neutrophilia, likely reflecting an ongoing inflammatory or infectious process (Table 1***)***.

**Table 1:** Patients’ laboratory data

Test	Result	Reference range
Biochemistry		
FBS (mg/dL)	492	70–115
BUN (mg/dL)	82	6.0–20.0
Creatinine (mg/dL)	1.8	0.5–1.00
Calcium (mg/dL)	8.6	8.6–10.3
Phosphorus	5.2	
RBC (10^6^/µl)	4.23	4.2–5.5
Hemoglobin (g/dL)	12.8	12–16
WBC (per µl)	18.200	4.000–11.000
MCV (fL)	88.2	80–99
Hematocrit (%)	37.3	37–47
Neutrophils (%)	89%	40–75
Lymphocytes (%)	1.3%	20–45
Eosinophils (%)	0%	0–6
MCH (pg/cell)	30.3	27–31
Troponin I (first sample)	Negative	Negative
Lipid profile, coagulation factors, and troponin		
Blood sugar (mg/dL)	492	74–106
LDH (IU/L)	594	235–470
Na (mEq/L)	123	135–148
K^+^ (mEq/L)	5.4	3.5–5.3
Creatinine (mg/dL)	1.8	0.5–1.00
CK-MB (U/L)	19	< 25
Troponin I (second sample in 6 hours)	Negative	Negative
BUN (mg/dL)	82	6.0–20.0
Calcium	8.6	8.6–10.3
Phosphorus	5.2	2.5–5.0
Total bilirubin	15.0	0.3–1.2
SGOT/AST	156	5–40
SGPT/ALT	468	0–40
Alk p	400	80–306
CPK	83	24–190
Total protein	6.9	6.0–7.8
Albumin	3.2	3.5–5.2
Amylase	20	0.1–100
D-dimer	504	< 500

*FBS* fasting blood sugar, *BUN* blood urea nitrogen, *RBC* red blood cell count, *WBC* white blood cell count, *MCV* mean corpuscular volume, *MCH* mean corpuscular hemoglobin, *LDH* lactate dehydrogenase, *Na* sodium, *K+* potassium, *CK-MB* creatine kinase-MB, *SGOT/AST* serum glutamic-oxaloacetic transaminase/aspartate aminotransferase, *SGPT/ALT* serum glutamic-pyruvic transaminase/alanine aminotransferase, *Alk p* alkaline phosphatase, *CPK* creatine phosphokinase

The patient’s cardiac and respiratory functions were immediately monitored in the ER. The electrocardiogram (ECG) showed no abnormalities. His previous medical records from about 2 weeks ago included abdominal ultrasonography showing a coarse-textured liver with irregular outlines (indicative of chronic parenchymatous liver disease); normal spleen, gallbladder, pancreas, and kidneys; no ascites or lymphadenopathy (LAP); and severe colonic gaseous distension.

**Fig. 1:**
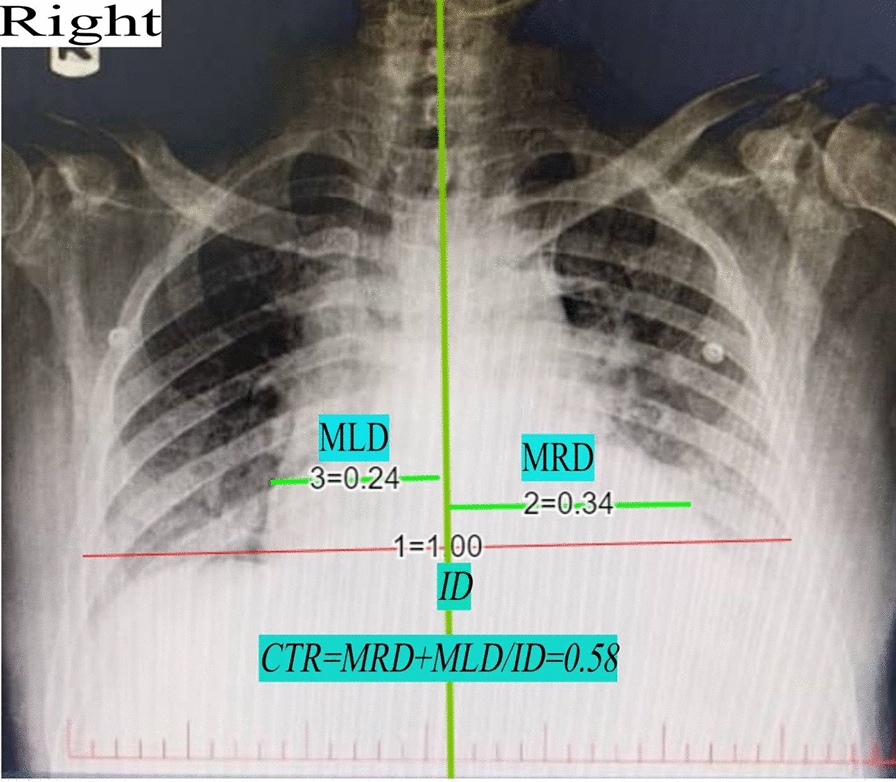
Chest X-ray of the patient with cardiac tamponade showing an enlarged, water bottle-shaped cardiac silhouette, indicative of significant pericardial effusion. *MLD* midline-left diameter, *MRD* midline-right diameter, *ID* internal diameter, *CTR* cardiothoracic ratio

The patient’s chest X-ray, which was taken last night in another medical center, revealed significant findings consistent with a higher than normal cardiothoracic ratio (0.58). The medical center had recommended admission for more investigation, but he had denied the suggestion and asked to be discharged. The X-ray (Fig. 1) demonstrated an enlarged cardiac silhouette, indicating a substantial pericardial effusion. This radiographic evidence highlighted the critical nature of the patient’s condition, necessitating immediate medical intervention.

The patient presented with signs and symptoms suggestive of cardiac tamponade, likely secondary to a severe pericardial effusion associated with advanced liver cirrhosis. Immediate transthoracic echocardiography was performed, which revealed a significant pericardial effusion of approximately 1100 mL, confirming the diagnosis of cardiac tamponade (Fig. 1). Considering the holistic results of laboratory tests, imaging studies, and physical examination findings all supported the diagnosis, with significant fluid retention and liver dysfunction. Owing to the patient’s critical condition and unstable vital signs, emergency pericardiocentesis was performed to relieve the tamponade and reduce intrapericardial pressure.

**Fig. 2:**
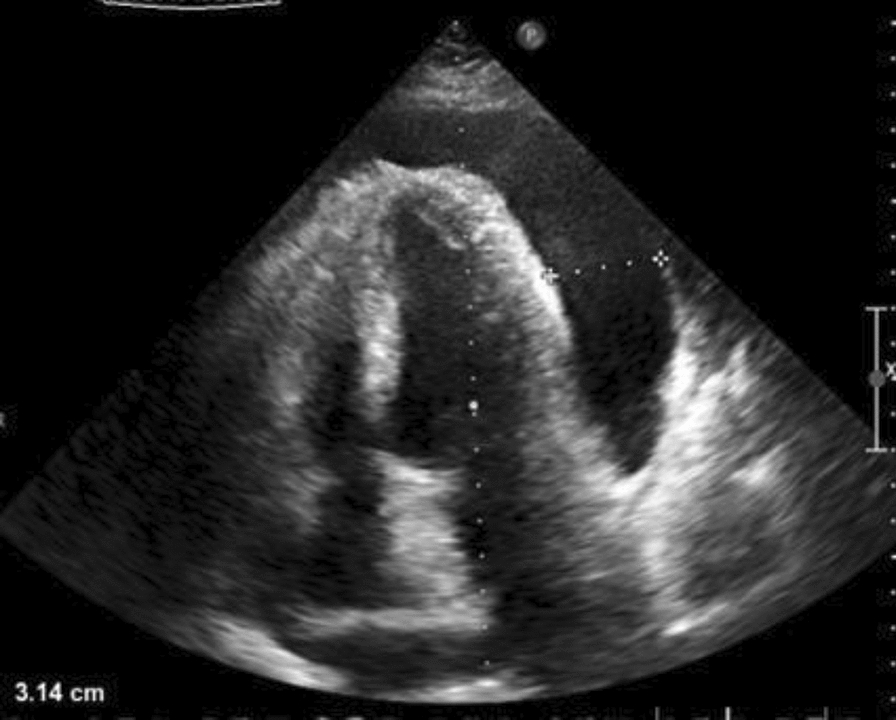
Transthoracic echocardiogram showed severe pericardial effusion (tamponade)

**Fig. 3:**
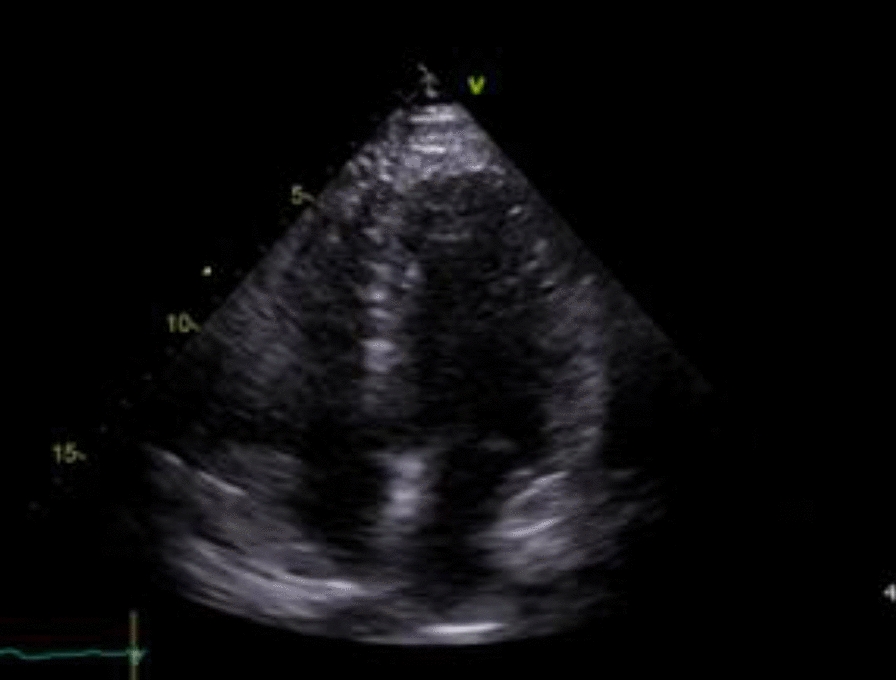
Post-procedural thoracocentesis transthoracic echocardiogram showed the resolution of tamponade

The emergency pericardiocentesis procedure was performed under sterile conditions and ultrasound guidance. A needle was inserted subxiphoid into the pericardial space, and approximately 1100 mL of serosanguinous fluid was aspirated (Fig. 2). The fluid was slightly reddish in color, indicating the presence of blood, likely owing to the inflammation and pressure effects on the pericardium. The fluid sample was sent to the laboratory for analysis, and the cardiac surgeon planned a pericardial window opening owing to the chronic nature of the disease and the high risk of recurrence of fluid accumulation. Following the procedure, ongoing monitoring and management included: continuous monitoring of vital signs; monitoring hemodynamic status; repeat TTE (Fig. 3) to assess pericardial effusion and cardiac function; close monitoring of liver and renal function; addressing electrolyte imbalances; and managing hyperglycemia.

The pharmacological management plan comprised several key components. Initially, furosemide was administered at 40 mg intravenously, followed by 20–40 mg intravenously every 6–8 hours to manage fluid overload. To manage autoimmune hepatitis and reduce inflammation, the patient was prescribed prednisone at a dosage of 40 mg orally daily. In continuation of his chronic treatment regimen for autoimmune hepatitis, azathioprine was administered at 50 mg orally daily. Additionally, ursodeoxycholic acid was included in the treatment plan at a dosage of 800 mg orally three times a day to improve liver function and manage cholestasis. To manage tachycardia and control HR, the patient was given metoprolol at 25 mg orally twice daily. To prevent gastrointestinal complications from corticosteroid therapy, omeprazole was prescribed at 20 mg orally daily. For managing hyperglycemia, insulin therapy was implemented using a sliding scale of regular insulin, starting with 2–4 units for every 50 mg/dL increment of blood glucose above 150 mg/dL, adjusted on the basis of blood glucose monitoring.

### Post-procedural follow-up

After being observed for 6 hours in the ER, the patient was admitted for further observation in the internal ward. A diagnostic coronary angiogram (CA) revealed no remarkable lesion except slow flow in the left anterior descending (LAD) and a dominant right coronary artery (RCA), recommending optimal medical therapy (Table 2). Computed tomography angiography (CTA) showed: normal pulmonary arteries down to small subsegmental levels with no emboli; marked pericardial effusion with an estimated 1100 mL of fluid; atelectatic bands and alveolar edema in the lower lobes; normal mediastinal vascular structures; no LAP or masses; a normal bronchial tree; minimal left pleural effusion; normal chest wall soft tissue with no focal lesions; normal bones with no focal lytic or sclerotic lesions; and a cirrhotic liver (Table 2).

**Table 2:** A comprehensive summary of the echocardiographic, computed tomography angiography (CTA), and coronary angiography (CA) findings, offering a detailed and structured presentation for this case report

Imaging modality	Findings
Transthoracic echocardiogram	
Ejection fraction	Preserved
Chamber sizes	Normal left and right ventricular size
Wall motion	No regional wall motion abnormalities
Pericardial effusion	Large pericardial effusion with evidence of tamponade physiology
Inferior vena cava	Dilated with minimal respiratory variation
Diastolic dysfunction	Present
Right atrium and ventricle	Evidence of right atrial and right ventricular diastolic collapse
Respiratory variation in mitral Inflow	Exaggerated respiratory variation in mitral inflow velocities
Computed tomography angiography	
Pulmonary arteries	Normal to small subsegmental levels with no emboli
Pericardial effusion	Marked pericardial effusion with an estimated 500 mL of fluid
Lung parenchyma	Atelectatic bands and alveolar edema in the lower lobes
Mediastinal structures	Normal mediastinal vascular structures
Lymph nodes	No lymphadenopathy or masses
Bronchial tree	Normal
Pleura	Minimal left pleural effusion
Chest wall	Normal chest wall soft tissue with no focal lesions
Bones	Normal bones with no focal lytic or sclerotic lesions
Liver	Cirrhotic liver
Coronary angiography	
Left main stem	No lesion
Left anterior descending	No critical lesion, slow flow
Left circumflex	No critical lesion
Right coronary artery	Dominant, no critical lesion
Left ventricular end-diastolic pressure	120/80 mmHg

The medical management plan included initiating optimal medical therapy per diagnostic CTA findings, considering diuretics to manage fluid overload, arranging consultations with cardiology specialists to manage further cardiac complications, and hepatology specialists for advanced liver disease management and potential liver transplantation evaluation. Follow-up plans involved regular short-term monitoring, including serial echocardiography to assess the resolution of the pericardial effusion, and frequent checks of vital signs, liver function, and renal function. Long-term follow-up focused on managing liver disease progression and preventing cardiac complications, with periodic liver imaging and continued use of ursodeoxycholic acid. Regular consultations with cardiology and hepatology specialists ensured comprehensive care, and the patient was periodically evaluated for liver transplantation eligibility. This multidisciplinary approach aimed to thoroughly and continuously manage the patient’s condition.

## Discussion

Cardiac tamponade can be either acute or chronic. Acute tamponade occurs rapidly and is often life-threatening, requiring immediate medical intervention. Chronic tamponade develops more slowly, allowing the pericardium to stretch and accommodate more fluid over time, but can still become a critical condition if not properly managed [13]. In this patient, the etiology of the severe pericardial effusion is likely multifactorial, with advanced liver disease and autoimmune hepatitis (AIH) playing significant roles.

Liver cirrhosis is a common consequence of chronic liver diseases, characterized by tissue fibrosis and the transformation of normal liver architecture into abnormal nodules [14]. The global prevalence of cirrhosis is not well-defined, but estimates suggest it is around 0.15% in the USA, with higher rates in Asia and Africa owing to chronic viral hepatitis [15, 16]. In another study, the global prevalence of liver cirrhosis in biopsy studies ranges from 4.5–9.5% of the world’s population [17]. Cirrhosis can lead to systemic complications such as ascites and pericardial effusion due to hypoalbuminemia, portal hypertension, and fluid retention [18]. The incidence of pericardial effusions in cirrhosis ranges from 32% to 63%, correlating with the degree of liver failure, and these effusions are typically small, resulting from the systemic effects of advanced liver disease [19]. The increased portal pressure from cirrhosis causes splanchnic vasodilation and activates vasoconstrictive pathways such as the renin–angiotensin–aldosterone system (RAAS), leading to fluid retention, ascites, and potentially pericardial effusion due to elevated hydrostatic pressure [20].

Additionally, hypoalbuminemia and impaired lymphatic drainage in patients with cirrhosis cause fluid leakage into the interstitial spaces and accumulation of lymphatic fluid in body cavities, including the pericardial sac, which may exacerbate pericardial effusion [18, 20, 21]. Common causes include chronic hepatitis C, heavy alcohol consumption, obesity-related non-alcoholic steatohepatitis, hepatitis B, hepatitis D, primary biliary cirrhosis, and AIH [14, 15]. According to literature, autoimmune hepatitis accounts for approximately 6% of all cases of cirrhosis [22].

AIH is a severe liver disease that can present either acutely or chronically, often with asymptomatic, insidious, or nonspecific symptoms. AIH affects individuals worldwide, across all ages and ethnicities, with a notable female predominance (75–80% of cases) regardless of the AIH subtype [23]. Diagnosis is based on clinical and laboratory criteria, including elevated serum transaminase and immunoglobulin G levels, specific circulating autoantibodies, and interface hepatitis on liver histology [24]. AIH typically arises in genetically predisposed individuals, especially women, when a viral infection triggers a T-cell-mediated autoimmune response against liver autoantigens. This response is facilitated by molecular mimicry (where immune responses to external pathogens target structurally similar self-proteins) and inadequate regulatory immune control, mainly owing to defects in regulatory T cells, leading to a loss of tolerance [25].

Impairment of immune regulation plays a central role in AIH. The condition responds well to immunosuppressive treatment, which should be initiated promptly. Standard regimens involve high initial doses of corticosteroids (prednisone or prednisolone), tapered as azathioprine is introduced. Liver transplantation is an excellent option for patients with acute liver failure or end-stage liver disease complications, including hepatocellular carcinoma, although AIH can recur or develop *de novo* post-transplantation [26]. Severe untreated AIH has a poor prognosis, but adequate treatment can significantly improve outcomes [27, 28]. This disease often requires long-term immunosuppressive therapy, which can predispose patients to infections and other complications [29]. Histologic findings of bridging necrosis or multilocular necrosis at presentation can progress to cirrhosis in 82% of untreated patients and are associated with a 5-year mortality of 45% [27, 28].

Inflammatory conditions like AIH can lead to pericarditis, an inflammation of the pericardium, resulting in pericardial effusion. This fluid buildup can compress the heart, leading to cardiac tamponade, a life-threatening condition [30–32]. Treatment of pericardial effusion may involve therapeutic pericardiocentesis, which immediately relieves symptoms by draining the accumulated fluid. In cases where pericardial effusion recurs or persists, surgical interventions such as pericardial window or pericardiectomy may be necessary to prevent fluid re-accumulation [33]. Cardiac tamponade occurs when rapid fluid accumulation in the pericardial sac increases intracardiac pressures, particularly in the right atrium, impeding normal cardiac filling. This rise in pressure can lead to cardiogenic shock and death, with symptoms such as elevated systemic venous pressure, occasional S4 gallop, and jugular venous distention [34]. The diagnosis of cardiac tamponade involves a combination of clinical examination and often bedside echocardiography [34, 35] and CT or MRI for detailed anatomical information and determining underlying causes [34]. Beck’s triad, consisting of hypotension, muffled heart tones, and neck vein distention, is considered diagnostic, but these signs may not always be present or easy to recognize [35]. The treatment for cardiac tamponade involves urgent pericardiocentesis to remove the fluid compressing the heart, often guided by echocardiography for precision. In severe cases, surgical interventions like creating a pericardial window or pericardiotomy are necessary to ensure proper drainage and prevent recurrence [36]. Mortality rates are higher in patients with tamponade; Queiroz *et al*. report a 31.5% mortality, significantly higher in those with tamponade [34].

According to the study by Fede *et al*., clinical outcomes for patients with pericardial effusion and cirrhosis significantly improve with early and appropriate management. The study highlights the importance of timely interventions such as pericardiocentesis in alleviating symptoms and preventing progression to cardiac tamponade, subsequently reducing mortality rates. Furthermore, addressing underlying causes, including liver disease and hypoalbuminemia, is crucial for preventing recurrence and enhancing overall prognosis. These findings emphasize the need for prompt and comprehensive management strategies in this patient population to optimize clinical outcomes [37].

Ziad Taimeh *et al*. (2012) emphasize the importance of early detection and treatment of pericarditis and pericardial effusion in patients with primary biliary cirrhosis (PBC) to prevent severe complications such as cardiac tamponade. They advocate for prompt interventions, such as pericardiectomy, and suggest that corticosteroids could be beneficial in managing the inflammatory nature of pericarditis in these patients [38]. This literature review summarizes cases of cardiac tamponade induced by cirrhosis, including patient demographics, clinical presentations, diagnostic findings, treatments, and outcomes (Table 3).

**Table 3:** Literature review of cirrhosis-induced cardiac tamponade

Author and YOP	Patient age/gender	Brief Hx, PH/E, Pos lab or imaging	Diagnosis, treatment/intervention, prognosis
Chung M.W. *et al*., 2018 [39]	56/Male	CC: dyspnea, chest discomfort, and low BP. Hx: cirrhosis due to alcohol abuse, COPD. MRI: 3.0 cm HCC in segment IVa.	Dx: PT post-RFA. Tx: pericardiocentesis. Prog: improved hemodynamics, discharged.
Pyarali F. *et al*., 2020 [40]	54/Male	CC: confusion. Hx: alcoholic cirrhosis and jaundice. PH/E: scleral icterus, jaundice, palmar erythema, gynecomastia, and distended abdomen. Lab: hyponatremia, elevated BUN and Cr. Echo: right ventricular collapse.	Dx: PT in cirrhosis and ascites. Tx: pericardiocentesis, PW. Prog: Improved post-drainage.
Chae M.S. *et al*., 2016 [41]	59/Male	Hx: liver cirrhosis, HBV, previous LT, diabetes, and HTN. PH/E: severe adhesions and hemorrhage during suprahepatic exploration. Lab: anemia, coagulopathy, and hyperlactatemia. Echo: showed 30 mm PE.	Dx: PT during liver retransplantation. Tx: pericardiotomy and sternotomy. Prog: stable post-operatively.
Taimeh Z. *et al*., 2012 [42]	49/Female	CC: fatigue and SOB. Hx: PBC on UDCA. PH/E: decreased breath sounds, distant heart sounds, no JVD. Lab: elevated liver enzymes and positive AMA. CT and Echo showed massive PE.	Dx: Pericarditis with massive PE in PBC. Tx: Pericardiectomy with PW. Prog: Improved respiratory status, stable on follow-up.
Cheung T.K. *et al*., 2004 [43]	41/Male	CC: fever. Hx: chronic HCV, cirrhosis, and diuretic-resistant ascites. PH/E: febrile, tense ascites left leg cellulitis. Lab: serum albumin 13 g/L, bilirubin 14 µmol/L. Echo showed large PE.	Dx: PE secondary to cirrhotic ascites. Tx: pericardiocentesis, PW, and orthotopic liver transplantation. Prog: no recurrence of effusions post-transplantation.
Islam S. *et al*., 1999 [44]	57/Female	CC: CP. Hx: PBC for 10 years, paranoid schizophrenia, and DM II. PH/E: pleuritic CP, fever, small bilateral PE. Lab: elevated liver enzymes, positive ANA. Echo showed large PE.	Dx: PT to SLE in PBC patient. Tx: pericardiocentesis, corticosteroids. Prog: improved; no more fluid after 2 weeks.

*AMA* antimitochondrial antibody, *ANA* anti-nuclear antibodies, *BP* blood pressure, *BUN* blood urea nitrogen, *CC* chief complaint, *COPD* chronic obstructive pulmonary disease, *CP* chest pain, *Cr* creatinine, *CT* computed tomography, *DM II* Diabetes Mellitus type 2, *Dx* diagnosis, *Echo* echocardiography, *HCC* hepatocellular carcinoma, *HBV* hepatitis B virus, *HCV* hepatitis C virus, *Hx* history, *HTN* hypertension, *JVD* jugular venous distention, *Lab* laboratory tests, *LT* liver transplantation, *MRI* magnetic resonance imaging, *PE* pericardial effusion, *PBC* primary biliary cirrhosis, *PH/E* physical examination, *Prog* prognosis, *PT* prothrombin time/pericardial tamponade, *PW* pericardial window, *RFA* radiofrequency ablation, *SOB* shortness of breath, *SLE* systemic lupus erythematosus, *Tx* treatment, *UDCA* ursodeoxycholic acid, *YOP* year of publication

## Conclusion and clinical key points

This case highlights that prompt recognition and comprehensive TTE and CTA evaluation are crucial in diagnosing cardiac tamponade. Emergency pericardiocentesis was lifesaving, underscoring the need for immediate intervention. A multidisciplinary approach is essential for managing such complex cases. Effective long-term management of autoimmune hepatitis and cirrhosis prevents recurrence and improves patient outcomes substantially.

## Data Availability

Data is available on request due to privacy/ethical restrictions.

## Abbreviations

AIH: Autoimmune hepatitis
IgG: Immunoglobulin G (IgG)
JVP: Jugular venous pressure
BUN: Blood urea nitrogen
ECG: Electrocardiogram
CTA: Computed tomography angiography
TTE: Transthoracic echocardiogram
LFT: Liver function tests
LAP: Lymphadenopathy
CA: Coronary angiogram
PBC: Primary biliary cirrhosis
FBS: Fasting blood sugar
RBC: Red blood cell count
WBC: White blood cell count
MCV: Mean corpuscular volume
MCH: Mean corpuscular hemoglobin
LDH: Lactate dehydrogenase
Na: Sodium
K^+^: Potassium
CK-MB: Creatine kinase-MB
SGOT/AST: Serum glutamic-oxaloacetic transaminase/aspartate aminotransferase
SGPT/ALT: Serum glutamic-pyruvic transaminase/alanine aminotransferase
Alk p: Alkaline phosphatase
CPK: Creatine phosphokinase
MLD: Midline-left diameter
MRD: Midline-right diameter
ID: Internal diameter
CTR: Cardiothoracic ratio
EF: Ejection fraction
IVC: Inferior vena cava
LMS: Left main stem
LAD: Left anterior descending
LCX: Left circumflex
RCA: Right coronary artery
LVEDP: Left ventricular end-diastolic pressure
ALT: Alanine aminotransferase
AMA: Antimitochondrial antibody
AST: Aspartate aminotransferase
BP: Blood pressure
BUN: Blood urea nitrogen
CC: Chief complaint
COPD: Chronic obstructive pulmonary disease
CP: Chest pain
Cr: Creatinine
CT: Computed tomography
Dx: Diagnosis
Echo: Echocardiography
HCC: Hepatocellular carcinoma
HBV: Hepatitis B virus
HCV: Hepatitis C virus
Hx: History
HTN: Hypertension
JVD: Jugular venous distention
Lab: Laboratory
LDH: Lactate dehydrogenase
LT: Liver transplantation
MRI: Magnetic resonance imaging
PE: Pericardial effusion
PH/E: Physical examination
Prog: Prognosis
PT: Prothrombin time/pericardial tamponade
PW: Pericardial window
RFA: Radiofrequency ablation
SOB: Shortness of breath
SLE: Systemic lupus erythematosus
Tx: Treatment
UDCA: Ursodeoxycholic acid
YOP: Year of publication
ANA: Anti-nuclear antibodies

## References

[1] Stashko E, Meer JMIS [Internet]. TI (FL): SP 2024 J. A from: Cardiac Tamponade. 2024.

[2] Meltser H, Kalaria VG. Cardiac tamponade. Catheterization Cardiovas Interv. 2005;64(2):245–55. 10.1002/ccd.20274 15678459

[3] Lee SS. Cardiac abnormalities in liver cirrhosis. West J Med. 1989;151(5):530–5. PMC1026787 2690463

[4] Wiegand J, Berg T. The etiology, diagnosis and prevention of liver cirrhosis. Dtsch Arztebl Int. 2013 10.3238/arztebl.2013.0085 PMC3583179 23451000

[5] Yoshiji H, Nagoshi S, Akahane T, Asaoka Y, Ueno Y, Ogawa K, *et al*. Evidence-based clinical practice guidelines for Liver Cirrhosis 2020. J Gastroenterol. 2021. 10.1007/s00535-021-01788-x. 34231046 10.1007/s00535-021-01788-x PMC8280040

[6] Shah A, Variyam E. Pericardial effusion and left ventricular dysfunction associated with ascites secondary to hepatic cirrhosis from the sections of cardiology. https://www.tarjomano.com/3341860

[7] Møller S, Danielsen KV, Bendtsen F. Pathophysiology behind cardiopulmonary complications of cirrhosis and portal hypertension. In: Milan Z, Goonasekera C, editors. Anesthesia for Hepatico-Pancreatic-Biliary Surgery and Transplantation. Springer International Publishing: Cham; 2020. p. 43–72.

[8] Jagdish RK, Roy A, Kumar K, Premkumar M, Sharma M, Rao PN, et al. Pathophysiology and management of liver cirrhosis: from portal hypertension to acute-on-chronic liver failure. Frontiers in Medicine. 2023. 10.3389/fmed.2023.1060073 PMC10311004 37396918

[9] Vergani D, Longhi MS, Bogdanos DP, Ma Y, Mieli-Vergani G. Autoimmune hepatitis. Semin Immunopathol. 2009;31(3):421–35. 19533129 10.1007/s00281-009-0170-7

[10] Hahn JW, Yang HR, Moon JS, Chang JY, Lee K, Kim GA, *et al*. Global incidence and prevalence of autoimmune hepatitis, 1970–2022: a systematic review and meta-analysis. EClinicalMedicine. 2023;1:65. 10.1016/j.eclinm.2023.102280 PMC10590724 37876996

[11] Trivedi PJ, Hirschfield GM. Recent advances in clinical practice: epidemiology of autoimmune liver diseases. Gut. 2021;1989:2003. 10.1136/gutjnl-2020-322362 34266966

[12] McFarlane IG. Definition and classification of autoimmune hepatitis. Semin Liver Dis. 2002;22(4):317–24. 12447704 10.1055/s-2002-35702

[13] Vakamudi S, Ho N, Cremer PC. Pericardial effusions: causes, diagnosis, and management. Prog Cardiovas Dis. 2017. 10.1016/j.pcad.2016.12.009. 10.1016/j.pcad.2016.12.009 28062268

[14] Pinzani M, Rosselli M, Zuckermann M. Liver cirrhosis. Best Pract Res Clin Gastroenterol. 2011;25(2):281–90. 21497745 10.1016/j.bpg.2011.02.009

[15] Schuppan D, Afdhal NH. Liver cirrhosis. The Lancet. 2008;371(9615):838–51. 10.1016/S0140-6736(08)60383-9 PMC2271178 18328931

[16] Bahardoust M, Dehkharghani MZ, Ebrahimi P, Najafirashed M, Mousavi S, Haghmoradi M, *et al*. Effect of ABO blood group on postoperative overall survival and recurrence-free survival rate in patients with hepatocellular carcinoma after hepatectomy: a multi-center retrospective cohort study. BMC Surg. 2023. 10.1186/s12893-023-02236-8. 37875876 10.1186/s12893-023-02236-8 PMC10599055

[17] Namazi A, Ebrahimi P, Sarveazad A, Khaleghian M, Bahardoust M, Mokhtare M, *et al*. Predictors for liver cirrhosis in patients with hepatitis C virus: a cross-sectional study. Hepat Mon. 2023. 10.5812/hepatmon-136164.

[18] Kashani A, Landaverde C, Medici V, Rossaro L. Fluid retention in cirrhosis: pathophysiology and management. An Int J Med. 2008;101:71–85. 10.1093/qjmed/hcm121 18184668

[19] Liu H, Lee SS. Cardiopulmonary dysfunction in cirrhosis. J Gastroenterol Hepatol. 1999;14(6):600–8. 10385072 10.1046/j.1440-1746.1999.01920.x

[20] Premkumar M, Anand AC. Overview of complications in cirrhosis. J Clin Exp Hepatol. 2022. 10.1016/j.jceh.2022.04.021. 35814522 10.1016/j.jceh.2022.04.021 PMC9257866

[21] Bendahmash A, Elsiesy H, Al-hamoudi WK. Cirrhotic ascites: pathophysiological changes and clinical implications. in: ascites - physiopathology, treatment, complications and prognosis. InTech; 2017.

[22] Ghadimi DJ, Ghorani H, Moradi Z, Golezar MH, Nouri S, Irilouzadian R, *et al*. Management of ectopic variceal bleeding with transjugular intrahepatic portosystemic shunt: a systematic review of case reports. Emergency Radiology. Springer Science and Business Media Deutschland GmbH; 2024. 10.1007/s10140-024-02258-6 38935315

[23] Mieli-Vergani G, Vergani D, Czaja AJ, Manns MP, Krawitt EL, Vierling JM, *et al*. Autoimmune hepatitis. Nat Rev Dis Primers. 2018;4(1):18017. 29644994 10.1038/nrdp.2018.17

[24] Heneghan MA, Yeoman AD, Verma S, Smith AD, Longhi MS. Autoimmune hepatitis. The Lancet. 2013. 10.1016/S0140-6736(12)62163-1. 10.1016/S0140-6736(12)62163-1 23768844

[25] Manns MP, Lohse AW, Vergani D. Autoimmune hepatitis-Update 2015. 10.1016/j.jhep.2015.03.005 25920079

[26] Manns MP, Czaja AJ, Gorham JD, Krawitt EL, Mieli-Vergani G, Vergani D, *et al*. Diagnosis and management of autoimmune hepatitis. Hepatology. 2010;51(6):2193–213. 20513004 10.1002/hep.23584

[27] Czaja AJ. Features and consequences of untreated type 1 autoimmune hepatitis. Liver Int. 2009;29(6):816–23. 19018980 10.1111/j.1478-3231.2008.01904.x

[28] Michielsen P, Francque, Vonghia, Ramon. Epidemiology and treatment of autoimmune hepatitis. Hepat Med. 2012. 10.2147/HMER.S16321. 24367228 10.2147/HMER.S16321 PMC3846915

[29] Mack CL, Adams D, Assis DN, Kerkar N, Manns MP, Mayo MJ, *et al*. Diagnosis and management of autoimmune hepatitis in adults and children: 2019 practice guidance and guidelines from the american association for the study of liver diseases. Hepatology. 2020. 10.1002/hep.31065. 31863477 10.1002/hep.31065

[30] Imazio M. Pericarditis: pathophysiology, diagnosis, and management. Curr Infect Dis Rep. 2011;13(4):308–16. 21534015 10.1007/s11908-011-0189-5

[31] Kontzias A, Barkhodari A, Yao Q. Pericarditis in systemic rheumatologic diseases. Curr Cardiol Rep. 2020;22(11):142. 10.1007/s11886-020-01415-w. 32910306 10.1007/s11886-020-01415-w

[32] Bizzi E, Trotta L, Pancrazi M, Nivuori M, Giosia V, Matteucci L, *et al*. Autoimmune and autoinflammatory pericarditis: definitions and new treatments. Curr Cardiol Rep. 2021;23(9):128. 10.1007/s11886-021-01549-5. 34319478 10.1007/s11886-021-01549-5

[33] Shah A, Variyam E. Pericardial effusion and left ventricular dysfunction associated with ascites secondary to hepatic cirrhosis from the sections of cardiology [Internet]. Available from: https://www.tarjomano.com 3341860

[34] de Queiroz CM, Cardoso J, Ramires F, Ianni B, Hotta VT, Mady C, *et al*. Pericardial effusion and cardiac tamponade: Etiology and evolution in the contemporary Era. Int J Cardiovasc Sci. 2021;34(5):24–31.

[35] Weiser TG. Cardiac Tamponade. 2024. Available from: https://www.msdmanuals.com/professional/injuries-poisoning/thoracic-trauma/cardiac-tamponade

[36] Stashko E, Meer JM. Cardiac tamponade continuing education activity. Available from: https://www.ncbi.nlm.nih.gov/books/NBK431090/

[37] Fede G, Privitera G, Tomaselli T, Spadaro L, Purrello F. Cardiovascular dysfunction in patients with liver cirrhosis. Ann Gastroenterol. 2015;28(1):31. 25608575 PMC4290002

[38] Taimeh Z, Hatfield C, Husainy R, Holthauser C, Pugh A, Dababneh H, *et al*. Pericarditis with massive pericardial effusion : an unusual complication of primary biliary cirrhosis. Acta Gastro-Enterologica Belgica. 23082708

[39] Chung MW, Ha SY, Choi JH, Park HJ, Myung DS, Cho SB, *et al*. Cardiac tamponade after radiofrequency ablation for hepatocellular carcinoma case report and literature review. Medicine. 2018. 10.1097/MD.0000000000013532. 30544457 10.1097/MD.0000000000013532 PMC6310525

[40] Pyarali F, Auerbach J, Cabrera J, Calderon Candelario RA. Cardiac tamponade - a rare complication of ascites and cirrhosis C43. Critical care case reports: the gastrointestinal tract, liver, and pancreas. 2020, A5142-A5142

[41] Chae MS, Jeon YK, Kim DG, Na GH, Yi YS, Park CS. Cardiac tamponade due to suprahepatic surgical exploration in liver retransplantation: a case report. Transplant Proc. 2016;48(9):3181–5. 27932177 10.1016/j.transproceed.2016.02.065

[42] Taimeh Z, Hatfield C, Husainy R, Holthauser C, Pugh A, Dababneh H, *et al*. Pericarditis with massive pericardial effusion an unusual complication of primary biliary cirrhosis. Acta Gastro-Enterol Belg. 2012;75(3):354. 23082708

[43] Cheung TK, Tam W, Bartholomeusz D, Harley H, Johnson R. Hepatic hydropericardium. J Gastroenterol Hepatol (Australia). 2004;19(1):109–12. 10.1111/j.1440-1746.2004.03128.x 14675253

[44] Islam S, Riordan JW, McDonald JA. Case report: a rare association of primary biliary cirrhosis and systemic lupus erythematosus and review of the literature. J Gastroenterol Hepatol. 1999;14(5):431–5. 10355507 10.1046/j.1440-1746.1999.01883.x

